# 19 years outcome after cementless total hip arthroplasty with spongy metal structured implants in patients younger than 65 years

**DOI:** 10.1186/s12891-016-1285-3

**Published:** 2016-10-18

**Authors:** Ludger Gerdesmeyer, Munjed Al Muderis, Hans Gollwitzer, Norbert Harrasser, Martin Stukenberg, Maria-Angela Clifford, Andreas Toepfer

**Affiliations:** 1 Klinik für Orthopädie und Unfallchirurgie, Universitätsklinik Schleswig Holstein, Arnold Heller Strasse 3, 24105 Kiel, Germany; 2 Sydney Adventist and Norwest Hospitals, University of New South Wales, 116 Macquarie Street, Parramatta, 2150 NSW Australia; 3 Klinik für Orthopädie und Sportorthopädie am Klinikum rechts der Isar der Technischen Universität München, Ismaningerstr.22, 81675 Munich, Germany; 4 Prince of Wales Private Hospital Sydney, Barker Street, Randwick, 2031 NSW Australia

**Keywords:** Total hip arthroplasty, Cementless, Spongy metal structured, Young patients

## Abstract

**Background:**

Cementless fixation of total hip arthroplasties (THAs) is often favored in young, high-demanding patients due to the conservation of valuable bone-stock and easier revision if loosening has occurred. Long-term outcome data of the spongy metal structured implant used in the present study in patients younger than 65 years are still lacking.

**Methods:**

We conducted a retrospective chart review and functional investigation (Merle d’Aubigné score, SF-12) of patients younger than 65 years at implantation treated with a spongy metal structured THA (*n* = 79) from one orthopedic university center from 1985 to 1989.

**Results:**

At a 19-year mean follow-up (range: 15.3 – 21.3 years), the overall stem survival rate was 93.7 %, and the overall cup survival rate was 82.3 %. Revision surgeries of the stem were performed in all cases for aseptic loosening at an average of 15.3 ± 3.5 years after implantation. Acetabular components were revised for aseptic loosening and recurrent dislocation after inlay revision on an average of 11.8 ± 4.7 years after implantation. No other device related complications occurred within the 19-year follow-up period. No correlation was found between time of revision and gender or age. Clinical outcome scores (Merle d’Aubigné score, SF-12) revealed excellent to good results of the implanted THAs in 87 % of patients.

**Conclusions:**

We conclude that spongy metal structured cementless THAs implanted in young patients have an excellent survival and provide trustworthy clinical results at 19 years of follow-up.

## Background

Total hip arthroplasty (THA) is one of the most successful surgical interventions ever developed. Results of cemented THAs showed lasting pain relief and a survivorship of more than 80 % at 20 years after surgery, however some negative predictive factors were identified that contribute to success or failure of the procedure [1]. In a 25-year follow-up study Berry et al. described that the survivorship free of revision for aseptic loosening was poorer for each decade earlier in life at which the procedure was performed [2]. They found that survivorship rate was 68.7 % in patients younger than 40 years compared to 100 % in patients aged 80 years and older. Males had a twofold higher rate of revision for aseptic loosening as compared to females.

**Fig. 1:**
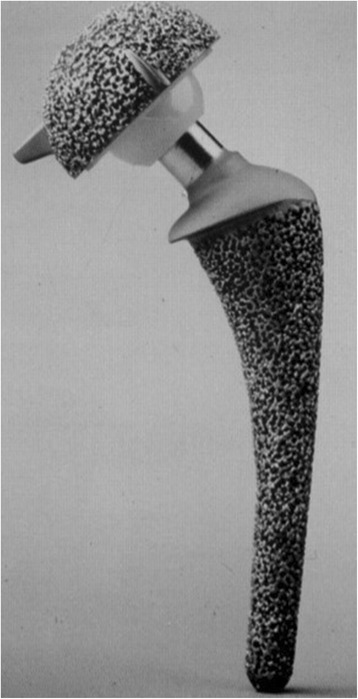
Spongy metal surface implant GHE

Technical evolution of cementless fixation implants with regards to surface structure, shape and metallurgic aspects has led to higher survival rates [3]. For long-term stability, bony anchorage of the implant should achieve specific parameters. Meso-structure surfaces in which pore size and depth copy cancellous bone morphology provide a unique feature on implants with direct bone contact [4]. Pore size of 100-2000 μm, and porosity larger than 40 % has been shown to favour osseous integration [5]. High form fit to the inner osseous structure and high primary stability with micro-movements less than 150 μm are also necessary for bony anchorage and high secondary long-term stability [6]. In this context implants have been developed with surface structures similar to that of human cancellous bone. By the use of a special wax molding process full porous implant surface cast in one piece with its core is achieved, allowing for a fully integrated core-surface structure (Fig. 1). This kind of integration allows for pore size to be between 800-1500 μm and depth reaching up to 3000 μm with an overall porosity of 60 % [4]. It also allows the formation of mesh spaces, forming an intercommunicating system extending throughout the surface of the implant.

The aim of the present study was to assess the long-term outcome in patients younger than 65 years after cementless THA with first generation spongy metal implants.

## Methods

This study was reviewed and approved by the Medical Ethical Committee at the Klinikum rechts der Isar (Technical University of Munich) and research was carried out in compliance with the Helsinki Declaration. Written informed consent was obtained from all patients included in the study.

One hundred consecutive cementless THAs were performed in 88 patients during the period between 1985 and 1989 using a spongy metal structured implant (ESKA/AQ Implants, stem-type *GHE*, Lübeck, Germany). All patients were prospectively enrolled in the study. Written informed consent was obtained from all patients. The mean age of the patients at time of surgery was 47 years (range: 18 to 65 years), with 51 males and 49 females out of 100 THAs. 49 right, and 51 left hip arthroplasties were performed.

**Fig. 2:**
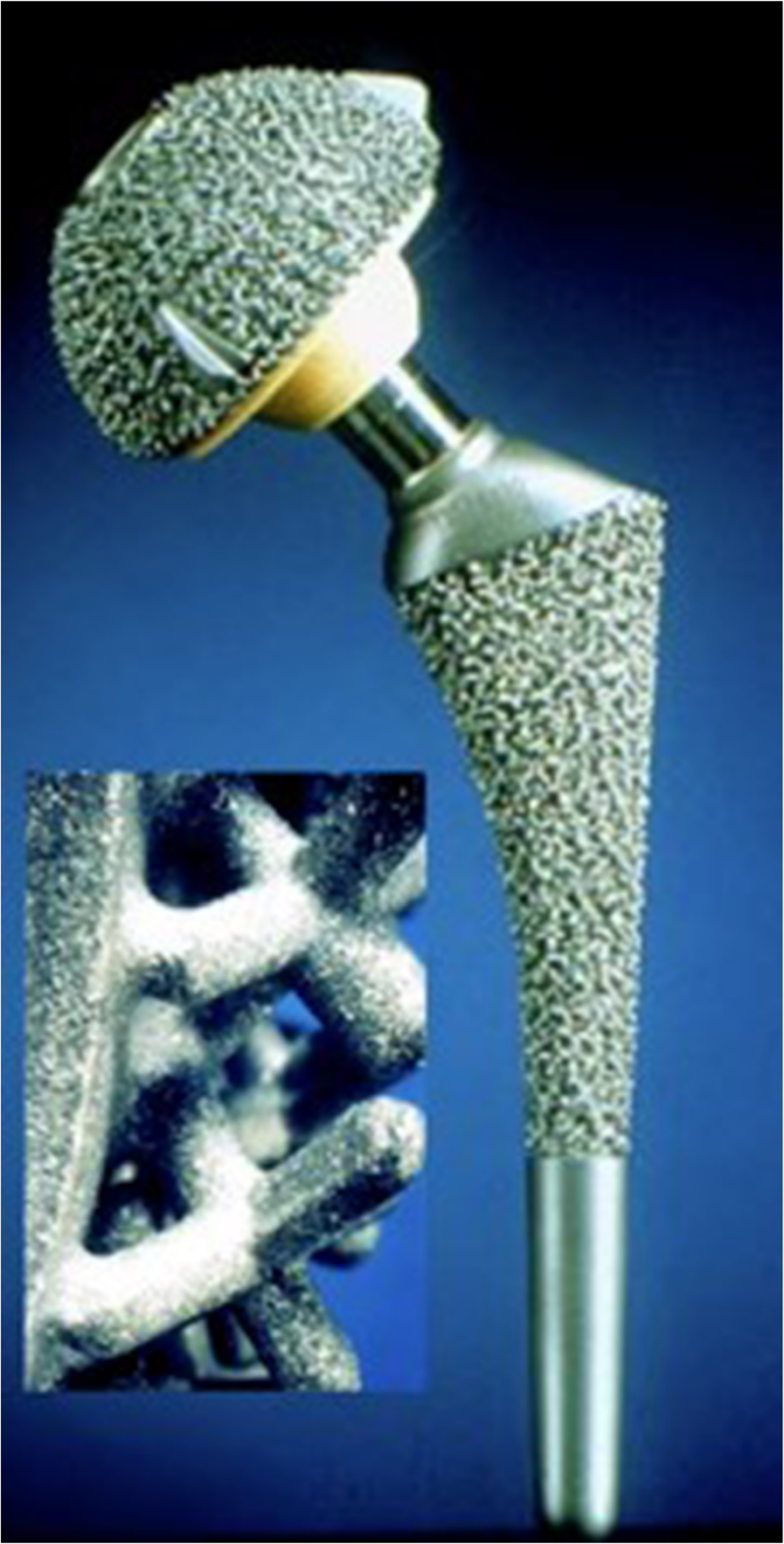
Modified GHE-implant with no collar and spongy metal surface (Spongiosa Metal®-II, see magnification) at the stem body and smooth surface at the distal portion

The implant used was an anatomically adapted, side specific anatomical double curved stem and hemispherical cup, with fully porous metal surface, that resembles cancellous bone (Fig. 1). The spongy metal structured surface has a porosity of 60 % and a pore size from 800-1500 μm. Two spikes wedged into the anterior and posterior parts of the acetabular rim and a peg embedded into the ischium assist in the initial stability of the metal socket. Minor changes of the stem design (removal of the collar, partially coated stem with new developed surface structure “Spongiosa Metal^®^-II”) were conducted after the inclusion period of the present study (Fig. 2). The GHE stem and metal socket of the implant are produced in a casting process using an alloy of chrome, cobalt and molybdenum. The metal backed articular surface is made of standard polyethylene. 28 mm ceramic heads were used in all patients. All components were sterilized using gamma irradiation in vacuum seal atmosphere.

All surgeries were performed in one specialized arthroplasty medical centre by senior surgeons using a posterior approach [7]. The patient was placed in a true lateral position, with the affected limb uppermost. A 10-15 cm curved incision across the buttock centered on the posterior aspect of the greater trochanter was performed. The gluteus maximus was split in the line of its fibers, crossing vessels were coagulated. The short external rotator muscles were dissected from their femoral insertion and retracted laterally so that the sciatic nerve was protected. The capsule was incised and the hip dislocated in maximum adduction and internal rotation. The femoral neck was resected to gain good access to the acetabulum. After placing the retractors, the acetabulum was reamed according to the desired diameter, the cup was placed with 40-40° inclination and 10-15° anteversion. To finish with the cup the inlay was inserted. Then the retractors were placed to gain good view to the femoral canal, and the stem was rasped to the desired size. Finally the stem was inserted and the final head placed after determining the exact leg length through dislocation tests with the provisional head in place.

Postoperatively, physiotherapy was conducted for the first 3 months. It consisted of exercises aimed for increasing circulation to the legs and feet to prevent thrombosis. Muscle strengthening and mobilization of the hip was a focus of the therapy. Patients were allowed to perform exercises in the water after removal of stitches. According to the past standard partial weight bearing within the first 6 weeks after surgery was allowed.

Patients were routinely followed 6-8 weeks postoperatively and then at final follow up. Due to the distance from the patients’ home and the clinic, not all patients were followed annually as it would be in accordance to the clinic’s standard.

Radiological evaluation was conducted of the operated side(s) in an anteroposterior and lateral view. X-rays were taken routinely immediately after implantation and at least at final follow-up visit. The radiographic criteria evaluated were bone atrophy, bone hypertrophy, radiolucent lines, osteolysis, and component subsidence [8]. A relative increase or decrease in cortical bone mass or density at latest follow-up versus the first postoperative radiographs was defined as bone hypertrophy or atrophy. Radiolucent lines were defined as lucencies at the bone-prosthesis interface with or without a sclerotic line. Osteolysis was defined as scalloping of bone that was not present on the first postoperative radiograph. Component subsidence was determined by measuring implant position relative to the tip of the greater trochanter (stem subsidence) or tear drop (cup subsidence) compared with that of the first postoperative radiograph. Deviation greater than 1 mm was considered significant.

Functional outcome was measured using the Merle d’Aubigné rating system [9]. Additionally, subjective clinical outcome was measured using MacNab’s outcome assessment of patient satisfaction [10]. The patient was asked to rate his general level of well-being after surgery: Excellent (no pain; no restriction of activity), Good (occasional hip pain of sufficient severity to interfere with the patient’s ability to do his normal work or his capacity to enjoy himself in his leisure hours), Fair (improved functional capacity, but handicapped by intermittent pain of sufficient severity to curtail or modify work or leisure activities), Poor (no improvement or insufficient improvement to enable increase in activities). Pain was scored on the 10-cm visual analogue scale (VAS). Furthermore, global outcome including mental and physical health was assessed by the SF-12 short form questionnaire. The mean follow-up time was 19 years (range 15.3-20.3 years) at the final assessment.

### Statistical analysis

Statistical evaluation and analysis were performed using SPSS 21.0 software (IBM SPSS, Armonk, NY). Survivorship analysis using Kaplan-Meier was carried out with revision for any reason as the end point. Statistical analysis of Merle d’Aubigné score and SF-12 was carried out using the paired *t*-test (significance level: 0.05). A power analysis revealed a total sample size of 26 (two independent study groups; dichotomous primary endpoint: failure vs. no failure; anticipated incidence of failure: 24 %; anticipated incidence of failure free survival: 76 %; alpha: 5 %; beta: 20 %; power: 80 %).

## Results

**Fig. 3:**
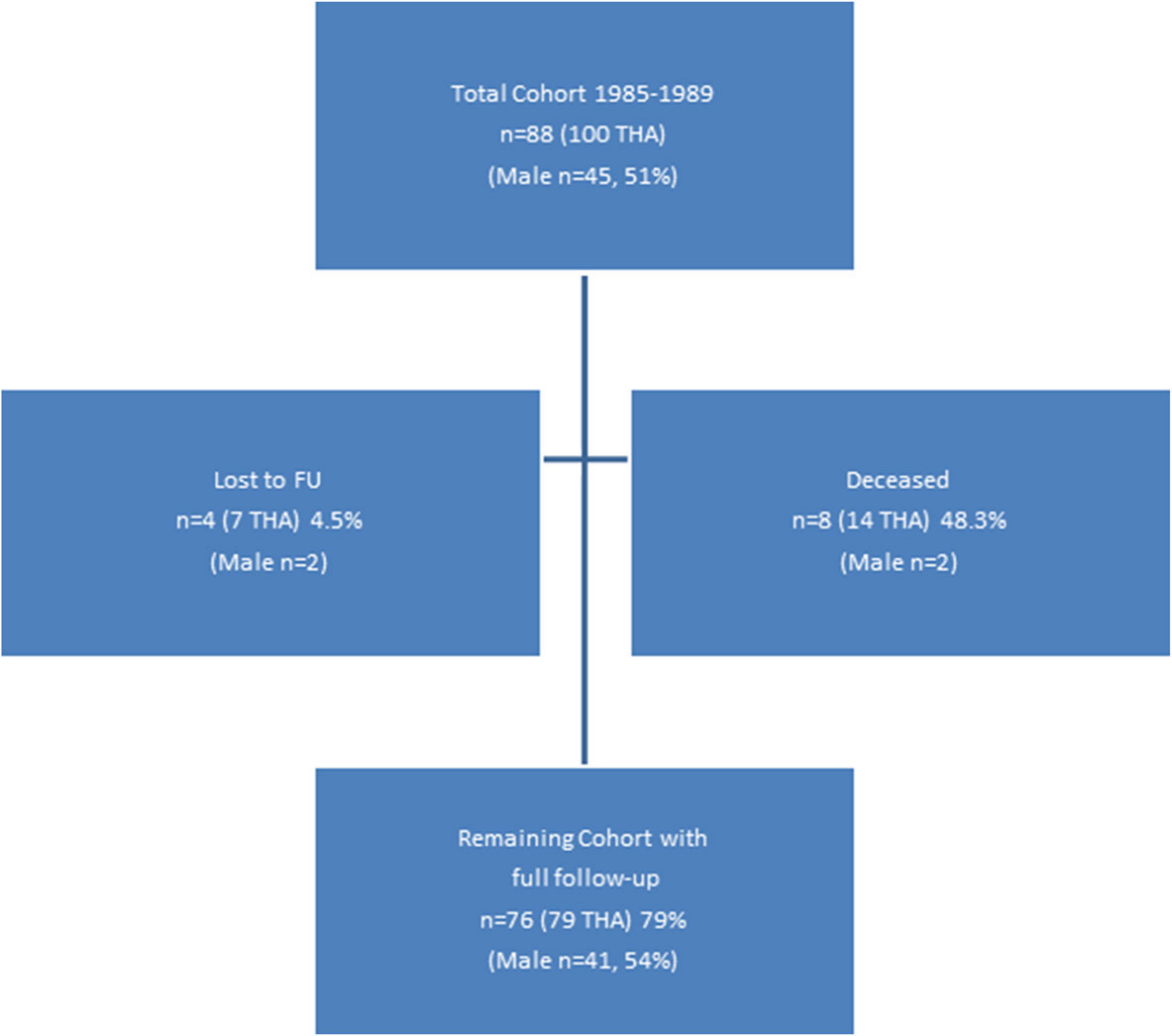
Patient distribution of the total cohort

At the time of the last follow-up 93 out of 100 hips could be evaluated (84 out of 88 patients). No data could be retrieved from 7 hips (4 patients). 2 patients were lost to follow-up (address unknown) and 2 subjects moved to other countries and refused to participate in the study. From the remaining 84 patients (93 hips) 8 patients (14 hips) had died without change or revision of the THA at 14.1 ± 4.3 years after surgery (Fig. 3).

79 remaining hips (76 patients, 41 males and 35 females) were enrolled in the final analysis (follow-up rate of 79 %). The mean age of the evaluated patients at the final follow-up was 64.8 ± 9.4 years (38 - 83 years) with a mean weight of 78.6 ± 17.0 kg (51 - 133 kg), mean height of 170.1 ± 8.9 cm (152 - 190 cm) and a mean body mass index (BMI) of 27.0 ± 4.4 kg/m^2^ (19.1 - 38.5 kg/m^2^). The diagnoses that had led to the THA are given in Table 1.

**Table 1:** Diagnoses leading to THA of study population

Diagnosis	Number of patients (%)
Hip Dysplasia	25 (32 %)
Epiphyseal Varus Deformity	14 (18 %)
Idiopathic Avascular Necrosis	12 (15 %)
Posttraumatic Avascular Necrosis	10 (13 %)
Rheumatoid Arthritis	3 (4 %)
Cortisone Induced Avascular Necrosis	3 (4 %)
Perthes Disease	3 (4 %)
Alcohol Induced Avascular Necrosis	2 (3 %)
Primary Osteoarthrosis	7 (9 %)

**Fig. 4:**
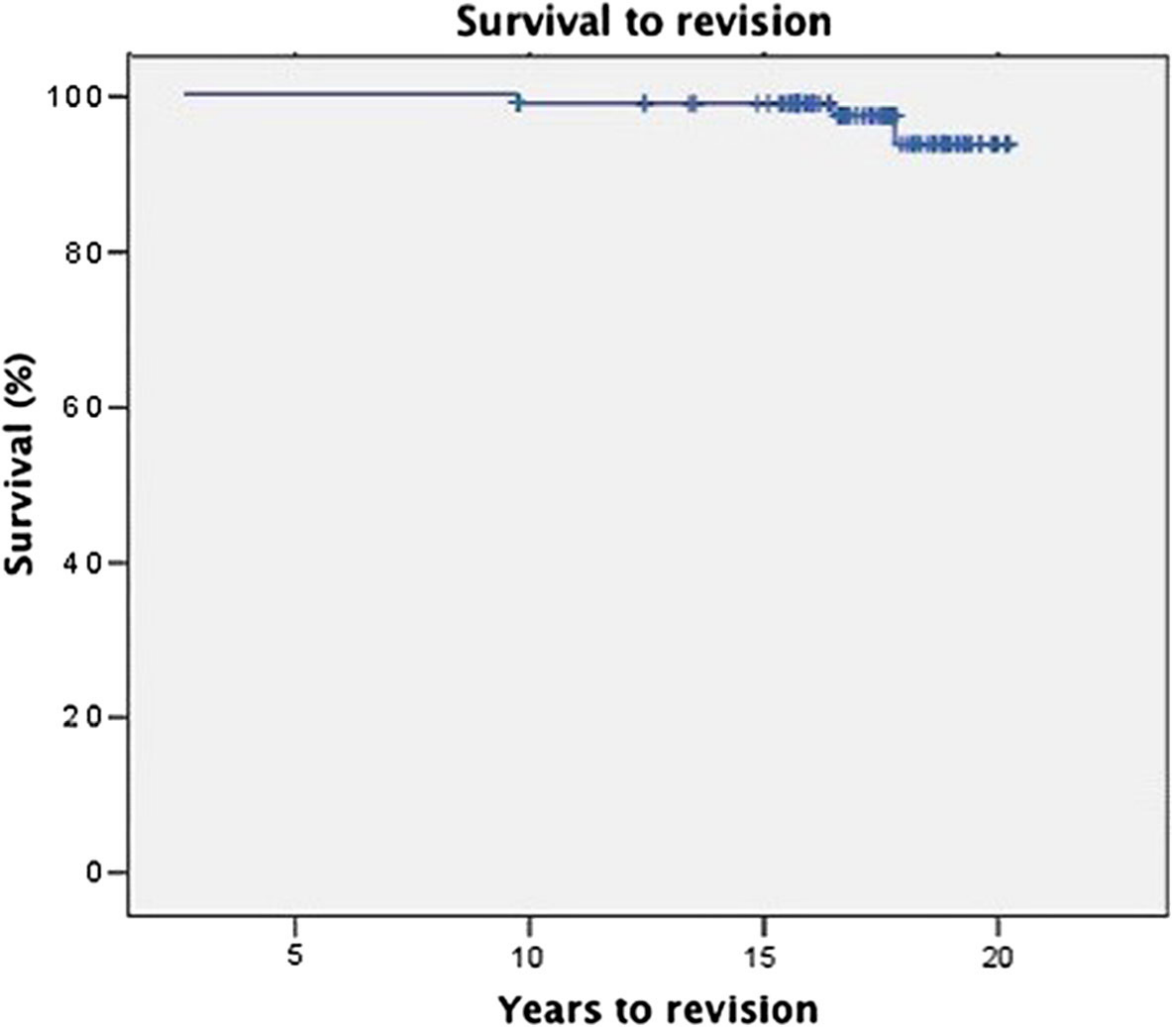
Kaplan-Meier survival curve of survival to stem-revision for any reason, with a survival of 93.7 % after 19 years of follow-up

**Fig. 5:**
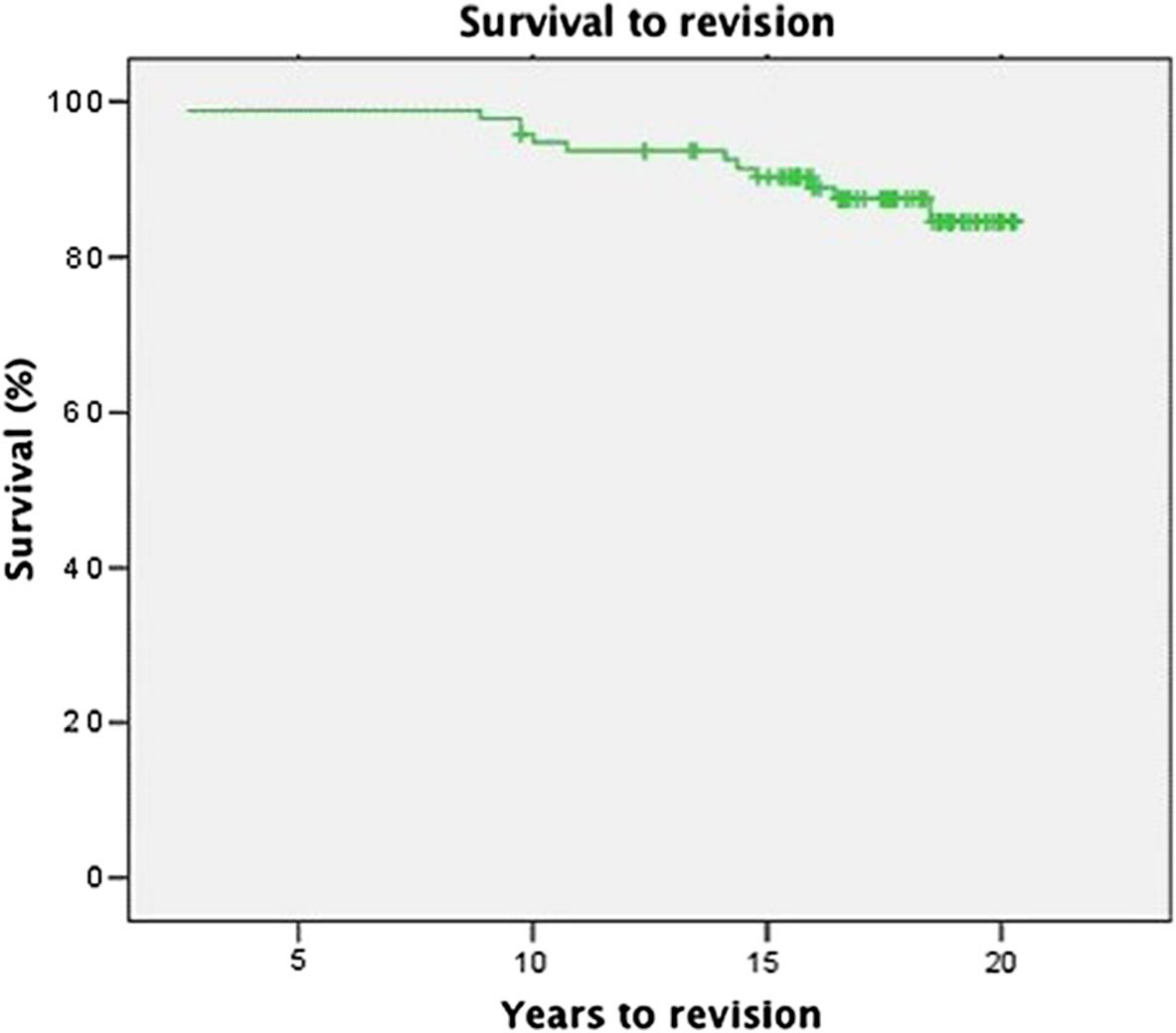
Kaplan-Meier survival curve of survival to socket-revision for any reason, with a survival of 82.3 % after 19 years of follow-up

The overall stem survival rate at follow-up was 93.7 % (Fig. 4). Revision of the stem was performed in 5 cases 15.3 ± 3.5 years after implantation (10.4 – 17.8 years) for aseptic loosening in all cases. 14 acetabular components were revised after 11.8 ± 4.7 years (2.6 – 18.5 years). Finally, the overall cup survival rate was 82.3 % after 19 years (Fig. 5). The reasons leading to revision of the socket were recurrent dislocation after inlay revision due to wear in 2 patients, aseptic loosening in 8 cases and a combined inlay and head revision was required due to wear in 4 hips. No other device related complications occurred within the 19-year follow-up period. No correlation was found between time of revision and gender or age.

Regarding radiological evaluation, all hips without revision showed signs of bone atrophy, bone hypertrophy, radiolucent lines, and osteolysis. No hip showed component subsidence, neither of the acetabular nor the femoral component.

The excellent results found in the Merle D’Aubigné Score (Table 2) were confirmed on the MacNab’s outcome assessment score. 19 years after THA, 60 % of the evaluated patients were classified as excellent, 27 % as good, 12 % as moderate and 1 % as poor. An excellent or good result was therefore observed in 87 %. Furthermore, 96 % of all patients would recommend the procedure of THA to a friend. Regarding clinical outcome scores, no differences were found relating to the patient’s gender.

**Table 2:** The functional outcome (Merle d’Aubigné) score regarding the item pain, mobility, and ability to walk

Item	Preoperatively	At final follow-up
Pain	2.70 ± 0.77	5.24 ± 1.15*
Mobility	3.59 ± 1.23	4.76 ± 1.32*
Ability to walk	3.19 ± 0.94 to	5.00 ± 1.32.*
Total score	9.47 ± 2.0	15.0 ± 3.1*

*p < .05

Evaluation of SF-12 questionnaires revealed a mean physical sum score of 45.74 (median 49.33), and a mean mental sum score of 50.24 (median 53.37). Thus, with respect to the mean physical sum score the patients were only marginally less healthy compared to a general US population (mean 50.0). The differences in SF-12 score values between patients with revision and without revision were not statistically significant.

## Discussion

Biological fixation of the implants by bone ingrowth is thought to be essential for the long-term success of a cementless THA. Stems which are porous-coated only in their proximal part have become popular. The more proximal stress transfer is believed to help prevent stress shielding, with less corrosion and release of metal ions, and easier removal of the implant if needed. There have been problems, however, with thigh pain and the early failure of the femoral component [11]. Even after there has been bone ingrowth into a limited part of the porous surface, partial porous coating may not provide sufficient stability. The spongy metal system examined in the present study provides excellent biologic anchorage due to the specific design of the structured surface. In contrast to porous coated surfaces, an interconnecting open structured design is used to achieve excellent structure compatibility. Porosity provides bony ingrowth but has to have a certain size which was calculated to be at least 40 % [4]. The second specification representing a significant impact on bony ingrowth is defined as porous size. If the pore size is less than 100 μm only soft tissue formation around the surface is observed, corresponding to elastic fibrous fixation of the implant. Several clinical studies found excellent survival rates if spongy metal structured implants were used. Matsui published a series of 49 patients in 1998 [12]. Excellent to good functional outcome was found in all patients after 5 to 9 years. No aseptic loosening was reported. Three out of 49 patients had thigh pain after surgery which disappeared within the first 2 years postoperatively. These mid-term results were confirmed by other authors [13]. On the other hand, long-term data regarding spongy metal structured implants are lacking. Therefore, the present study is the first to show high survival rates and excellent to good functional results in the long-term follow-up 19 years after surgery. Overall GHE stem survival was 93.7 % (Fig. 4), acetabulum component survival was 82.3 % (Fig. 5). Additionally, we could not find any correlation between clinical outcome and BMI. Similar results were reported by McLaughlin et al. who found no statistically significant difference between obese and non-obese patients with regards to clinical and radiological outcome or complications [14].

Our results suggests that use of cementless spongy metal fixation in primary THA in young patients is associated with low implant failure rates. On the other hand, there is only limited evidence regarding this topic. A meta-analysis of 20 studies using stratified and regression methods found no difference between fixation of THA with and without cement when revision of either component or both components was used as the study end-point. The sub-group analysis however demonstrated a trend toward superior results in younger patients who were managed with cementless fixation [15]. In studies of patients aged less than 45 years at the time of implantation, White et al. reported a revision rate of 14 % at 7.5 years [16]. Dorr et al. reported a revision rate of 19 % at only 4.5 years, with 45 % of the surviving implants showing radiologic signs of impending failure [17]. Sharp and Porter reported a 14 % revision rate at 6 years in patients aged less than 40 years and implicated rheumatoid arthritis as the main cause of loosening [18]. Williams and McCullough reported loosening in 24.6 % of hips at 4.5 years, but in 43.5 % of implants that had been in place longer than 5 years [19]. A large cross sectional study of Charnley-THA was published by Allami et al. [20]. The authors demonstrated a cumulative survival rate of 93.1 % at 10 years, the overall outcome was therefore comparable to those from the Swedish and Norwegian joint registries. Recent data from joint registries support these findings (Table 3) [21].

**Table 3:** Distribution of fixation methods (cementless: uncementd THA; cemented: cup and stem cemented; hybrid: cup cementless, stem cemented; reverse hybrid: cup cemented, stem cementless) according to country, and 10-year age- and fixation-matched survival analysis based on registry data

	Cementless	Cemented	Hybrid/reverse hybrid
Registry	Fixation [%]		
Australia	64	5	31
Sweden	15	68	17
Denmark	68	16	16
Norway	20	53	27
New Zealand	45	14	41
England and Wales	46	33	21
Spain	70	10	20
Canada	83	1	16
Age (years)	10 years Survival-rate [%]		
<65	92.2	93.5	93.5
65-7	93.1	94.1	94.2
>75	91.2	94.1	94.3

Focusing on the acetabulum site, fixation technique is discussed controversially. Callaghan et al. have reported the long-term results of cemented THAs with 13 % aseptic loosening at 19 years [22]. The aseptic loosening rate increases up to 52 % if the population younger than 50 years is reviewed [23]. The poor results of cemented acetabular components prompted many surgeons to change technique to cementless implants with biologic fixation and acetabular bony ingrowth. Marshall et al. eported on a cementless system series [24]. Seventy-four hips underwent revision of the acetabular component. Of these 74 % were threaded acetabular components and 26 % had a porous hemispherical cup with adjuvant screws. Midterm results showed significant improvement over cemented acetabular shells. The authors conclude that lower rates of aseptic loosening provides a scientific justification to favour biologic fixation for the acetabular component, especially in younger patients.

The optimal method to obtain femoral fixation remains controversial as well. In contrast to the acetabulum site the results of cemented and cementless femoral fixation are comparable. Excellent long-term results have been observed for cemented and cementless stems [1, 25]. Poor short-term results have been observed for both methods of fixation as well [15]. A significant risk factor in this context are high demanding, active patients. In this context, a study reports revision rates of 34 % (socket) and 23 % (stem) if a cemented Charnley-THA-System was used in patients younger than 30 years [26]. Alternative methods of fixation have therefore been explored in this population [27].

## Conclusion

In summary, our findings of the excellent long-term outcome after primary spongy metal surface THA in young patients demonstrate the effectiveness of cementless implants if required specifications are respected.

## Abbreviations

BMI: Body mass index
THA: Total hip replacement
VAS: Visual analogue scale
